# Double product reflects the association of heart rate with MACEs in acute coronary syndrome patients treated with percutaneous coronary intervention

**DOI:** 10.1186/s12872-017-0714-z

**Published:** 2017-12-02

**Authors:** Tan Xu, Youqin Zhan, Nan Lu, Zhuoqiao He, Xi Su, Xuerui Tan

**Affiliations:** 1 Department of Cardiology, First Affiliated Hospital of Shantou University Medical College, Changping Road NO.57, Shantou, Guangdong 515041 China; 2 Department of Cardiology, Wuhan Asian Heart Hospital, Wuhan, Hubei 430022 China

## Abstract

**Background:**

There is little information about the prognostic value of double product (DP) for acute coronary syndrome (ACS) patients treated with percutaneous coronary intervention (PCI). The aim of this study was to investigate whether DP reflects the predictive power of heart rate (HR) or systolic blood pressure (SBP) in ACS patients treated with PCI.

**Methods:**

A total of 7590 ACS patients who had undergone PCI, free from cardiac shock, were included. The follow-up duration was two years. The main adverse cardiovascular events (MACEs) included all-cause death, recurrent myocardial infarction and stroke.

**Results:**

In the unadjusted model, significantly higher rates of MACEs were recorded in the high DP group (relative risk 1.41, 95%CI 1.08 to 1.83, *p* = 0.012). However, in the full adjusted models, after including HR and SBP, the predictive value of DP was not significant (relative risk 0.86, 95%CI 0.55 to1.33, *p* = 0.499). The predictive value of HR for MACEs was statistically significant (relative risk 1.74, 95% CI 1.33–2.28, *p* < 0.001). It was worth noting that the history of hypertension was strongly associated with MACEs (relative risk 1.53, 95% CI 1.11–2.11, *p* = 0.009).

**Conclusion:**

High DP is associated with MACEs for ACS patients treated with PCI. However, the predictive value of DP weakened when adjusted for HR. Therefore, we have shown that DP may reflect the predictive power of HR for ACS patients treated with PCI.

**Electronic supplementary material:**

The online version of this article (10.1186/s12872-017-0714-z) contains supplementary material, which is available to authorized users.

## Background

It has been widely recognized that high blood pressure, especially high systolic blood pressure (SBP), is a risk factor for cardiovascular diseases [1]. Some studies have shown that heart rate (HR) is also a risk factor for mortality and cardiovascular morbidity in patients with acute coronary syndrome (ACS) [2]. Recently, our meta-analysis concluded that elevated HR may increase the mortality of ACS patients in the percutaneous coronary intervention era, irrespective of admission, rest or discharge HR [3].

Double product (DP), which includes both the values of SBP and HR, was initially calculated to indirectly assess myocardial oxygen uptake during stress testing [4, 5]. DP has been demonstrated as a predictive parameter to evaluate prognosis in acute myocardial infarction patients treated with thrombolytic agents [6]. However, in the general population, one study found that DP did not have any value in predicting mortality in addition to SBP and HR [7].

To date, there is little information about the prognostic value of DP for ACS patients who were treated with percutaneous coronary intervention (PCI). This study aimed to investigate whether DP reflects the predictive ability of HR or SBP in ACS patients treated with PCI.

## Methods

### Study population

A total of 7824 ACS patients who had undergone PCI between January 2011 and December 2014 at Wuhan Asian Heart Disease Hospital, Wu Han, China were included. This is a tertiary hospital that has performed over ten thousand coronary intervention procedures.

The diagnosis of ACS, which includes ST elevated myocardial infarction and non-ST elevated acute coronary syndrome, was based on the standard guidelines [8, 9]. After admission, patients received reperfusion therapy with PCI. Appropriate medication strategies were used according to the practical guidelines of ACS management [8, 9].

### Study design

This is a retrospective study of prospectively collected data. The primary source of data was the hospital electronic medical record database and the follow-up database, which contains doctors’ performance records. The electronic medical record database included baseline demographics, clinical manifestation, procedure details and complications. The information in the follow-up database was mainly based on telephone and clinic visits.

Written informed consent was not obtained from participants because the primary source of data was collected for clinical audits and evaluation of our services. However, this study was approved by Wuhan Asian Heart Disease Hospital Ethics Committee for data extraction and acquisition from the hospital databases.

### Data collection

Patient characteristics, including gender, age, height, weight and medical history (hypertension, diabetes mellitus and dyslipidaemia) were collected at baseline. Patients were determined to be hypertensive if any one of the following conditions was present: SBP was 140 mmHg or higher, diastolic blood pressure was over 90 mmHg or use of antihypertensive medication [10]. Type 2 diabetes mellitus was diagnosed according to the criteria of the American Diabetes Association as a self-reported diagnosis of diabetes, plasma fasting glucose ≥7.0 mmol/L (or 2-h postprandial glucose ≥11.1 mmol/L), or use of diabetes medication at admission [11]. Dyslipidaemiawas defined according to the guidelines or use of statins [12]. BMI was defined as the body weight divided by the square of the body height.

HR and blood pressure at admission were measured accurately at the first medical contact by physicians using calibrated mercury sphygmomanometers. DP was defined as the product of SBP (mmHg) and HR (beats per minutes).

The follow-up duration was two years, during which the occurrence of main adverse cardiovascular events (MACEs) was recorded. The MACEs included all-cause death, recurrent myocardial infarction and stroke. All outcome analyses only considered the first occurrence of an event. All outcome information was extracted from the follow-up database.

### Statistical analysis

Categorical variables are presented as percentages. Chi-square tests were performed for categorical variables. Continuous variables are presented as the mean and standard deviation. The continuous variables, such as age, BMI, SBP, diastolic blood pressure, and HR, were compared using Mann–Whitney tests because they were all not normally distributed.

Because there is no established optimal threshold for DP, receiver operator characteristics (ROC) curve analysis was performed to assess the predictive ability of DP and HR on the MACEs. The point where sensitivity and specificity were maximized was determined as the best cut-off point.

We used Cox proportional hazards regression analysis to estimate the relative risk of two-year MACEs. Models for DP and HR were initially adjusted for fundamental biological characteristics, which were age (quartile), gender and body mass index (BMI) (quartile). The fully adjusted models for DP included age (quartile), gender, BMI (quartile), history of hypertension, dyslipidaemia, diabetes mellitus, HR (quartile) and SBP (quartile). The fully adjusted models for HR included age (quartile), gender, BMI (quartile), history of hypertension, dyslipidaemia, diabetes mellitus and SBP (quartile). Interactions between DP, HR and SBP were tested by adding interaction terms to the model.

A *p*-value of 0.05 was considered statistically significant. All statistical analyses were performed using SPSS version 16.0 (SPSS Inc., Chicago, Illinois, USA).

## Results

### Baseline characteristics

Of the 7824 patients, 214 patients were excluded because of incomplete data. In addition, 20 patients with cardiogenic shock at admission were excluded because of their labile haemodynamic parameters.

A total of 7590 consecutive ACS patients were included in this study. The mean age was 60.13 ± 9.76 years, and 73.4% of them were males. In this population, 64.43% had hypertension, 26.63% had diabetes mellitus, and 24.63% had known dyslipidaemia. The majority of patients (75.59%) were admitted with a non-ST-elevation acute myocardial infarction.

The ROC analysis showed that a D*P* value of 9657 was the cut-off with highest sensitivity and specificity in terms of prognostic significance. DP > 9657 was considered the high DP group, and DP ≤ 9657 was considered the low DP group. The population clinical characteristics were grouped by dichotomy of DP, which was based on the ROC cut-off (Table 1).

### Incidence of MACE events

During the two years of follow-up, 124 (1.56%) patients died, including 103 cardiovascular deaths and 21 from another cause of death. Fifty-four patients experienced non-fatal recurrent myocardial infarctions; and forty-eight had non-fatal strokes. In total, the incidence rate of MACEs was 2.94%. MACEs occurred in 94(2.45%) patients with DP < 9657 and 129(3.43%) patients with DP > 9657.

### Double product

**Fig. 1:**
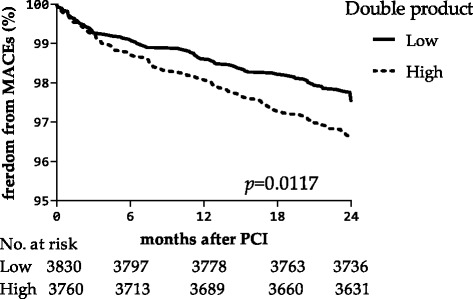
Kaplan-Meier curve showing the risk of MACEs, stratified by double product (*p* = 0.0117, cut-off9657mmHg*bpm). The number of patients at risk at the beginning of each half year is shown below the horizontal axis

In the unadjusted model, the high DP group (DP > 9657) had significantly higher rates of MACEs than the low DP group (DP ≤ 9657) (relative risk (RR) 1.41, 95% confidence interval (CI) 1.08 to 1.83, *p* = 0.012) (Fig. 1a). In multivariate Cox regression models that only adjusted for fundamental biological characteristics, high DP was also a significant predictor for MACEs (RR 1.36, 95%CI 1.04 to 1.78, *p* = 0.022). However, in the full adjusted models, after including HR and SBP, DP was not significant (RR 0.86, 95%CI 0.55 to1.33, *p* = 0.499) (Table 2). Interestingly, HR (treated with quartile) was significantly associated with MACEs (RR 1.75, 95%CI 1.083 to 2.817, *p* = 0.022). Therefore, we analysed heart rate separately in the following. Although the relationship between SBP and MACEs was not statistically significant in the fully adjusted model, it is important to note that history of hypertension was a risk factor for MACEs in this population (RR 1.54, 95% CI 1.118 to 2.115, *p* = 0.008). However, there was no significant association between DP and mortality.

**Table 1:** Population clinical characteristics stratified by dichotomy of DP based on ROC cut-off (9657 mmHg* bpm)

Characteristic	All	Dichotomy of double product		*p* value
	*n* = 7590	Low(*n* = 3830)	High(*n* = 3760)	
Male, n (%)	5559(73.24)	2902(75.77)	2657(70.66)	<0.001
Age (year)	60.13 ± 9.73	59.60 ± 9.73	60.66 ± 9.71	<0.001
BMI (kg/m^2^)	24.84 ± 3.20	24.63 ± 3.17	25.05 ± 3.22	<0.001
STEMI, n (%)	1853(24.41)	1012(26.42)	841(22.37)	<0.001
Diabetes mellitus, n (%)	2021(26.63)	866(22.61)	1155(30.72)	<0.001
Hypertension, n (%)	4890(64.43)	2139(55.85)	2751(73.16)	<0.001
Dyslipidemia, n (%)	1958(24.63)	937(24.46)	1021(27.15)	<0.001
Heart rate (bpm)	73.23 ± 11.98	66.58 ± 7.75	80.01 ± 11.72	<0.001
SBP (mmHg)	129.77 ± 18.74	118.89 ± 13.65	140.86 ± 16.60	<0.001
DBP (mmHg)	78.22 ± 11.24	73.37 ± 9.31	83.15 ± 10.90	<0.001
DP (mmHg* bpm)	9525.70 ± 2201.88	7874.43 ± 955.85	11,208.73 ± 1800.17	<0.001

*DP* double product, *ROC* receiver operator characteristics, *BMI* body mass index, *STEMI* ST-segment elevated myocardial infarction, *SBP* systolic blood pressure, *DBP* diastolic blood pressure, *bpm* beats per minutes

*means multiply by

**Table 2:** Full-adjusted multivariate analysis using Cox proportional hazards regression testing the relation between double product and two-year MACEs

Characteristic	Relative risks	95% CI	*P* value
DP	.859	0.554–1.333	0.499
Gender	1.142	.846–1.541	.387
hypertension	1.537	1.118–2.115	.008
Diabetes mellitus	1.308	.981–1.743	0.067
Dyslipidemia	.893	.646–1.233	0.492
SBP			0.605
Q1(<120 mmHg)	reference		
Q4(>140 mmHg)	.786	.498–1.240	.301
Heart rate			0.012
Q1(<66 bpm)	reference		
Q4(>80 bpm)	1.746	1.083–2.817	.022
Age			0.000
Q1(<54 years)	reference		
Q4(>67 years)	3.017	2.017–4.512	.000
Body mass index			0.155
Q1(<23 kg/m^2^)	reference		
Q4(>26 kg/m^2^)	.647	.438–0.957	.029

*MACEs* main adverse cardiovascular events, *DP* Double product, *SBP* systolic blood pressure, *Q1* first quartile, *Q4* fourth quartile

### Heart rate

**Fig. 2:**
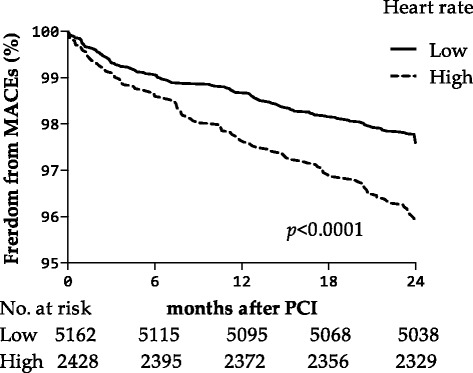
Kaplan-Meier curve showing the risk of MACEs, stratified by heart rate (*p* < 0.0001, cut-off 76 beats per minute). The number of patients at risk at the beginning of each half year is shown below the horizontal axis

A HR value of 76 was determined as the best cut-off. The population was grouped by dichotomy of HR (threshold as 76 beats per minute) into a high HR group and low HR group. In the unadjusted model, significantly higher rates of MACEs were found in the high HR group than in the low HR group (relative risk 1.71, 95%CI 1.31 to 2.23, *p* < 0.001) (Fig. 2).

Initially, the multivariate Cox model was adjusted by gender, sex, age and BMI. High HR was also a significant predictor for MACEs (RR 1.76, 95%CI 1.35 to 2.28, *p* < 0.001). In the fully adjusted model, history of hypertension, diabetes mellitus and dyslipidaemia, and SBP were included (Table 3). The predictive value of HR for MACEs was statistically significant (relative risk 1.74, 95% CI 1.33–2.28, *p* < 0.001). As in the DP model, there was no significant association between SBP and MACEs. It was worth noting that the history of hypertension was strongly associated with MACEs (relative risk 1.53, 95% CI 1.11–2.11, *p* = 0.009). No significant association was found between HR and mortality.

**Table 3:** Full-adjustedmultivariate analysis using Cox proportional hazards regression testing the relation between heart rate and two-year MACEs

Characteristic	Relative risks	95% CI	*p*value
Heat rate	1.744	1.334–2.279	0.000
Gender	1.138	.843–1.537	0.397
Diabetes mellitus	1.307	.980–1.742	.068
hypertension	1.530	1.112–2.105	.009
Dyslipidemia	.887	.642–1.225	.467
SBP			0.703
Q1(<120 mmHg)	reference		
Q4(>140 mmHg)	.868	.601–1.254	.451
Age			0.000
Q1(<54 years)	reference		
Q4(>67 years)	2.993	2.003–4.474	.000
Body mass index			0.144
Q1(<23 kg/m^2^)	reference		
Q4(>26 kg/m^2^)	.642	.434–0.950	.027

*MACEs* main adverse cardiovascular events, *SBP* systolic blood pressure, *Q1* first quartile, *Q4* fourth quartile

## Discussion

This is the first study investigating the predictive ability of DP for ACS patients. Retrospective evaluation of DP in ACS patients with two years of follow up yielded the following salient findings. The main conclusions were as follows: (i) high DP, which was calculated from the product of admission HR and SBP, is associated with MACEs; (ii) after adjusting for HR and SBP, the predictive ability of DP disappeared. However, HR was strongly associated with MACEs, which suggests that DP reflects the predictive ability of HR in ACS patients over the two years of follow-up. (iii) Additionally, history of hypertension was associated with increased risk of two-year MACEs, but not the level of systolic blood pressure SBP, in both the DP and HR models.

Initially, DP aimed to indirectly estimate myocardial oxygen uptake during exercise stress testing [4, 5]. Villella and colleagues demonstrated that DP was a predictive index to evaluate prognosis in survivors of acute myocardial infarction treated with thrombolytic agents able to perform an exercise test after acute myocardial infarction [6]. Their results showed that low DP was significantly associated with a higher 6-month mortality rate (RR 1.71, *p* = 0.020) [6]. However, in our analysis, low admission DP was a protective factor for two-year MACEs. The DP in our study was derived from the admission HR and SBP in the acute phrase of ACS patients. This may explain the inconsistency between the two studies. Overall, these results show the reliability of DP as a predictor.

In Japanese Ohasama populations, the DP at rest based on home blood pressure measurement was significantly associated with mortality [13]. Notably, the association between the home-measured DP and mortality was stronger than that between mortality and SBP or HR [13]. The results of the Ohasama study were partially consistent with our study. Although both revealed that high DP was associated with adverse events, the predictive value of DP in our study disappeared after adjusting for HR and SBP. This discrepancy may be due to differences in study population, outcomes and measurement methods of biomarkers. All participants in Ohasama study were from a Japanese population without a history of cardiovascular disease, and the DP was evaluated based on the home-measured SBP and HR. Nevertheless, all participants in our study were ACS patients, and the DP was measured based on admission SBP and HR in the acute phrase.

Recently, a study of 9937 participants in the general population demonstrated that DP reflects the predictive power of SBP, as the DP was not associated with mortality after adjusting for SBP [7]. These results were completely opposite from those of the aforementioned Ohasama study [13] but partially in agreement with ours. Overall, the role of DP has been seriously questioned in previous studies-, especially in different populations.

In addition, the TAIST trial reported that a high DP in the acute phase of stroke can predict poor outcome [14]. However, there is no evidence regarding the predictive value of DP for ACS patients after PCI.

As reported, admission SBP and HR have been demonstrated as predictors for in-hospital mortality and long-term mortality [2, 15–17]. Currently, some ACS risk models, such as PURSUIT [18] and GRACE [19], have also included admission HR as a prognostic factor. DP, which takes into account both SBP and HR, was assumed to be a strong predictor for ACS patients in our study. Unfortunately, the results from our study suggest that DP was not useful after HR and SBP were used to predict MACEs in ACS patients because the predictive value of DP was weakened after the model was adjusted for other haemodynamic parameters, such as HR. HR has been demonstrated as an independent risk factor of MACE events in ACS patients, as we have reviewed in a meta-analysis [3].

In our study, there was no significant relationship between SBP and two-year MACEs, which was inconsistent with previous study [15, 16]. This may be due to the exclusion of cardiogenic shock patients and in-hospital MACEs. Our results showed that DP or HR were not associated with mortality, which may be due to the low incidence of mortality in our population. However, our study revealed that history of hypertension is a risk factor of MACE in ACS patients, as reported in previous studies [20, 21].

### Study limitations

The present study must be interpreted within the context of its potential limitations. First, this is a retrospective observational analysis with inherent flaws in study design, although the data were prospectively collected and stored. Second, information about medication prior to hospital admission was not available, which may partly influence the HR and SBP. Third, the follow-up was mainly based on telephone interviews, which may bias events.

## Conclusion

In conclusion, our data suggest that DP is associated with main adverse cardiovascular events for ACS patients before adjustment for HR. However, the association of DP with MACEs disappeared when adjusted for HR. Therefore, we showed that DP may reflect the predictive ability of HR. Overall, among ACS patients treated with PCI, a history of hypertension is associated with two-year MACEs, not the level of SBP.

## Additional file

Additional file 1: Raw data. (SAV 400 kb)

## Abbreviations

ACS: Acute coronary syndromes
DP: Double product
HR: Heart rate
MACEs: Main adverse cardiovascular events
PCI: Percutaneous coronary intervention
SBP: Stolic blood pressure

## References

[1] Lewington S, Clarke R, Qizilbash N, Peto R, Collins R (2002). Age-specific relevance of usual blood pressure to vascular mortality: a meta-analysis of individual data for one million adults in 61 prospective studies. Lancet.

[2] Noman A, Balasubramaniam K, Das R, Ang D, Kunadian V, Ivanauskiene T, Zaman AG (2013). Admission heart rate predicts mortality following primary percutaneous coronary intervention for ST-elevation myocardial infarction: an observational study. Cardiovasc Ther.

[3] Xu T, Zhan Y, Xiong J, Lu N, He Z, Su X, Tan X (2016). The relationship between heart rate and mortality of patients with acute coronary syndromes in the coronary intervention era: meta-analysis. Medicine.

[4] Kitamura K, Jorgensen CR, Gobel FL, Taylor HL, Wang Y (1972). Hemodynamic correlates of myocardial oxygen consumption during upright exercise. J Appl Physiol.

[5] Gobel FL, Norstrom LA, Nelson RR, Jorgensen CR, Wang Y (1978). The rate-pressure product as an index of myocardial oxygen consumption during exercise in patients with angina pectoris. Circulation.

[6] Villella M, Villella A, Barlera S, Franzosi MG, Maggioni AP (1999). Prognostic significance of double product and inadequate double product response to maximal symptom-limited exercise stress testing after myocardial infarction in 6296 patients treated with thrombolytic agents. GISSI-2 investigators. Grupo Italiano per lo studio della Sopravvivenza nell-Infarto Miocardico. Am Heart J.

[7] Schutte R, Thijs L, Asayama K, Boggia J, Li Y, Hansen TW, Liu YP, Kikuya M, Bjorklund-Bodegard K, Ohkubo T (2013). Double product reflects the predictive power of systolic pressure in the general population: evidence from 9,937 participants. Am J Hypertens.

[8] Wright RS, Anderson JL, Adams CD, Bridges CR, Casey DE, Jr., Ettinger SM, Fesmire FM, Ganiats TG, Jneid H, Lincoff AM et al: 2011 ACCF/AHA focused update of the guidelines for the Management of Patients with unstable angina/non-ST-elevation myocardial infarction (updating the 2007 guideline): a report of the American College of Cardiology Foundation/American Heart Association task force on practice guidelines developed in collaboration with the American College of Emergency Physicians, Society for Cardiovascular Angiography and Interventions, and Society of Thoracic Surgeons. J Am Coll Cardiol 2011, 57(19):1920–1959. 10.1016/j.jacc.2011.02.009 21450428

[9] Kushner FG, Hand M, Smith SC, Jr., King SB, 3rd, Anderson JL, Antman EM, Bailey SR, Bates ER, Blankenship JC, Casey DE, Jr. et al: 2009 Focused updates: ACC/AHA guidelines for the Management of Patients with ST-elevation myocardial infarction (updating the 2004 guideline and 2007 focused update) and ACC/AHA/SCAI guidelines on percutaneous coronary intervention (updating the 2005 guideline and 2007 focused update): a report of the American College of Cardiology Foundation/American Heart Association task force on practice guidelines. Circulation 2009, 120(22):2271–2306. 10.1161/CIRCULATIONAHA.109.192663 19923169

[10] Chobanian AV, Bakris GL, Black HR, Cushman WC, Green LA, Izzo JL, Jr., Jones DW, Materson BJ, Oparil S, Wright JT, Jr. et al: The seventh report of the joint National Committee on prevention, detection, evaluation, and treatment of high blood pressure: the JNC 7 report. JAMA 2003, 289(19):2560–2572. 10.1001/jama.289.19.2560 12748199

[11] Report of the expert committee on the diagnosis and classification of diabetes mellitus. Diabetes Care 2003, 26 Suppl 1:S5–20. 10.2337/diacare.26.2007.s5 12502614

[12] Catapano AL, Reiner Z, De Backer G, Graham I, Taskinen MR, Wiklund O, Agewall S, Alegria E, Chapman MJ, Durrington P (2011). ESC/EAS guidelines for the management of dyslipidaemias: the task force for the management of dyslipidaemias of the European Society of Cardiology (ESC) and the European atherosclerosis society (EAS). Atherosclerosis.

[13] Inoue R, Ohkubo T, Kikuya M, Metoki H, Asayama K, Kanno A, Obara T, Hirose T, Hara A, Hoshi H (2012). Predictive value for mortality of the double product at rest obtained by home blood pressure measurement: the Ohasama study. Am J Hypertens.

[14] Sprigg N, Gray LJ, Bath PM, Boysen G, De Deyn PP, Friis P, Leys D, Marttila R, Olsson JE, O'Neill D (2006). Relationship between outcome and baseline blood pressure and other haemodynamic measures in acute ischaemic stroke: data from the TAIST trial. J Hypertens.

[15] Ma WF, Liang Y, Zhu J, Yang YM, Tan HQ, LT Y, Gao X, Feng GX, Li JD (2016). Comparison of 4 admission blood pressure indexes for predicting 30-day mortality in patients with ST-segment elevation myocardial infarction. Am J Hypertens.

[16] Bangalore S, Qin J, Sloan S, Murphy SA, Cannon CP (2010). What is the optimal blood pressure in patients after acute coronary syndromes?: relationship of blood pressure and cardiovascular events in the PRavastatin OR atorVastatin evaluation and infection therapy-thrombolysis in myocardial infarction (PROVE IT-TIMI) 22 trial. Circulation.

[17] Antoni ML, Boden H, Delgado V, Boersma E, Fox K, Schalij MJ, Bax JJ (2012). Relationship between discharge heart rate and mortality in patients after acute myocardial infarction treated with primary percutaneous coronary intervention. Eur Heart J.

[18] Boersma E, Pieper KS, Steyerberg EW, Wilcox RG, Chang WC, Lee KL, Akkerhuis KM, Harrington RA, Deckers JW, Armstrong PW (2000). Predictors of outcome in patients with acute coronary syndromes without persistent ST-segment elevation. Results from an international trial of 9461 patients. The PURSUIT investigators. Circulation.

[19] Granger CB, Goldberg RJ, Dabbous O, Pieper KS, Eagle KA, Cannon CP, Van De Werf F, Avezum A, Goodman SG, Flather MD (2003). Predictors of hospital mortality in the global registry of acute coronary events. Arch Intern Med.

[20] Majahalme SK, Smith DE, Cooper JV, Kline-Rogers E, Mehta RH, Eagle KA, Bisognano JD (2003). Comparison of patients with acute coronary syndrome with and without systemic hypertension. Am J Cardiol.

[21] Ali WM, Zubaid M, El-Menyar A, Al Mahmeed W, Al-Lawati J, Singh R, Ridha M, Al-Hamdan R, Alhabib K, Al Suwaidi J (2011). The prevalence and outcome of hypertension in patients with acute coronary syndrome in six middle-eastern countries. Blood Press.

